# High-resolution surface water dynamics in Earth’s small and medium-sized reservoirs

**DOI:** 10.1038/s41598-022-17074-6

**Published:** 2022-08-12

**Authors:** Gennadii Donchyts, Hessel Winsemius, Fedor Baart, Ruben Dahm, Jaap Schellekens, Noel Gorelick, Charles Iceland, Susanne Schmeier

**Affiliations:** 1 Deltares, Delft, The Netherlands; 2 Planet Labs PBC, Haarlem, The Netherlands; 3 Google, Zürich, Switzerland; 4 World Resources Institute, Washington, DC USA; 5 IHE Delft, Delft, The Netherlands; 6 Delft University of Technology, Delft, The Netherlands

**Keywords:** Environmental sciences, Hydrology, Climate sciences

## Abstract

Small and medium-sized reservoirs play an important role in water systems that need to cope with climate variability and various other man-made and natural challenges. Although reservoirs and dams are criticized for their negative social and environmental impacts by reducing natural flow variability and obstructing river connections, they are also recognized as important for social and economic development and climate change adaptation. Multiple studies map large dams and analyze the dynamics of water stored in the reservoirs behind these dams, but very few studies focus on small and medium-sized reservoirs on a global scale. In this research, we use multi-annual multi-sensor satellite data, combined with cloud analytics, to monitor the state of small (10–100 ha) to medium-sized (> 100 ha, excluding 479 large ones) artificial water reservoirs globally for the first time. These reservoirs are of crucial importance to the well-being of many societies, but regular monitoring records of their water dynamics are mostly missing. We combine the results of multiple studies to identify 71,208 small to medium-sized reservoirs, followed by reconstructing surface water area changes from satellite data using a novel method introduced in this study. The dataset is validated using 768 daily in-situ water level and storage measurements (r2 > 0.7 for 67% of the reservoirs used for the validation) demonstrating that the surface water area dynamics can be used as a proxy for water storage dynamics in many cases. Our analysis shows that for small reservoirs, the inter-annual and intra-annual variability is much higher than for medium-sized reservoirs worldwide. This implies that the communities reliant on small reservoirs are more vulnerable to climate extremes, both short-term (within seasons) and longer-term (across seasons). Our findings show that the long-term inter-annual and intra-annual changes in these reservoirs are not equally distributed geographically. Through several cases, we demonstrate that this technology can help monitor water scarcity conditions and emerging food insecurity, and facilitate transboundary cooperation. It has the potential to provide operational information on conditions in ungauged or upstream riparian countries that do not share such data with neighboring countries. This may help to create a more level playing field in water resource information globally.

## Introduction

Over 50% of water extracted for agriculture, domestic and industrial production globally comes from surface water^1^, of which most comes from reservoirs. Furthermore, most renewable energy worldwide comes from hydropower^2^, which is primarily dependent on reservoir storage. Reservoirs are operated by entities such as hydropower companies, particularly for large reservoirs, but also local or regional Water Users Associations^3,4^, agriculture companies or associations or other local private or public entities. Although reservoirs and the way they are controlled affect many in society, information on the location, size, and operations of dams or the level of reservoirs is scarce. Because reservoirs are generally operated via control schemes that are not publicly available, they are usually challenging to simulate, particularly in real-time and at a global scale, without in-situ data. The lack of near real-time information on the dynamics of reservoirs may result in: suboptimal use of water and operation of reservoirs in the hydropower and agriculture sector, disagreements between sectors such as agriculture and hydropower over the use of water and the related operation of reservoirs, lack of response to sudden-onset disasters such as dam flood releases and dam breaks, and distrust, the spread of (perceived) misinformation and conflicts between upstream and downstream users, possibly from other riparian countries^5,6^.

The operations of reservoirs can heavily impact the environment and societies^2,7^ and may even have geopolitical implications. This is particularly problematic as information on the operation of reservoirs remains one of the least shared types of water data. This is an issue, especially in transboundary basins. International water law at the global level, e.g., the 1997 UN Watercourses Convention, calls for data and information sharing between riparian states sharing a watercourse. Additionally, specific provisions for data sharing have been developed at the basin level (e.g., through international water treaties), requiring riparian states to share data and information on specific dimensions of the shared watercourse^8–10^. These requirements hardly ever include sharing data on the operation of individual dams, however. A lack of such transboundary data and information sharing presents a challenge to cooperative and sustainable basin management, particularly in regions with strong or rising conflict potential over shared water resources^5,11^. According to the Transboundary Freshwater Dispute Database (TFDD)^12^, dam development is one of the most common reasons for conflict between states, having the highest ratio of conflict compared to cooperation (50%).

The era of publicly available satellite data opens doors to global-scale monitoring of controlled water bodies, democratizing water resources information^13^. Currently, planetary-scale, high-resolution environmental monitoring is feasible using the Google Earth Engine (GEE) platform^14^, as illustrated by advances in worldwide monitoring of forests^15^, shorelines^16^, water bodies^17,18^, and rivers^19^. Applications such as Global Forest Watch^20^ demonstrate that near real-time monitoring is also feasible. The first global studies focusing on monitoring of water levels, surface water area, and storage changes of reservoirs using satellite data included the use of radar altimetry and MODIS satellites^21,22^. Numerous studies have also been published focusing on the estimation of water dynamics in reservoirs. However, to our knowledge, existing global studies mostly focus on large water bodies or on the use of data from a single satellite mission^23–28^. GEE was recently used to reconstruct long surface area time series for 428 reservoirs, including medium-sized water bodies using the Landsat archive and a topology infilling method for cloud-covered areas^26^. With the start of NASA’s ICESat-2 mission, it is now also possible to monitor water levels of smaller surface water bodies. Cooley et.al^27^ used ICESat-2 to study water level variations of over 200,000 water bodies worldwide. This revealed that surface water level variability to the largest degree is due to human-managed reservoirs. The available observation period (2018-now) of ICESat-2 is still too short for capturing trends in water levels for small reservoirs and how climate variability affects the water availability in these lakes and reservoirs.

Here we show that thanks to lake, reservoir^29,30^, and dam^31^ inventories and planetary-scale multi-mission remote sensing, small and medium-sized reservoirs across the globe can be monitored in near real-time. We present trends and variability in the surface area of 71,208 small to medium-size water bodies worldwide by processing nearly 35 years (~ 80 million images) of satellite data globally using GEE. In summary, we find that smaller reservoirs are much more sensitive to climate variability than large ones. Differentiating small reservoirs (0.1–1 km^2^) and medium-sized reservoirs (1–100 km^2^), we see that the variability of smaller-sized reservoirs tends to be larger than that of medium-sized reservoir water bodies. The inter-annual variability of small reservoirs is 52% higher than that of medium-sized reservoirs. More importantly, the intra-annual variability is 84% higher. These differences in intra-annual and inter-annual variabilities may imply that communities that rely on small-sized reservoirs are more vulnerable to water scarcity than those located near larger ones, both at seasonal and annual time scales. Only values measured after 2000 were used to compute inter-/intra-annual statistics due to the fact that Landsat observation frequency varies significantly geographically in early Landsat missions.

We advocate that the monitoring of reservoirs be operationalized at high spatio-temporal resolution (10 m in space and weekly in time). We demonstrate the potential uses of such monitoring via several possible examples, including claims regarding water scarcity, drought early warning, response, and food insecurity; and transboundary water conflicts and cooperation. We also demonstrate that our satellite analysis can be operationalized to near real-time and that all use cases are viable with automated operations.

## Results

### Mapping and global statistics

We have for the first time established and applied analytics to large amounts of satellite data, to monitor a total of 71,208 small to medium-sized reservoirs (sizes varying from 0.01 to 100 km^2^), with a revisit frequency of at least one week (though the actual observation frequency varies depending on the satellite image availability and quality). For reference, we consider a size of 10 ha to be small, similar to the minimum size of lakes in the HydroLAKES database^31^. We use a combination of remote sensing image processing algorithms on multi-petabyte satellite datasets and cloud analytics through the GEE platform. Recent work on mapping dams and reservoirs so far has focused on the identification of dams and reservoirs^30,31^. Studies focusing on higher-resolution surface water dynamics of reservoirs at a global scale have mainly researched a smaller number of reservoirs^25,26^. In our study, we combine data from multiple Landsat and Sentinel satellites to ensure revisit times are short enough to assess the status of reservoirs. We collate a set of reservoirs in the form of geospatial polygons from several dam and waterbody datasets, and per reservoir establish image-to-image surface water area estimates over non-cloudy areas, followed by a filling of remaining occluded pixels using the probability of surface water occurrence^18^. The entire method is described in the Methods section. We perform validation for four countries (Spain, India, South Africa and the USA) and 768 reservoirs in total, resulting in high goodness of fit between satellite-derived surface water area dynamics and daily in-situ water level and storage measurements with the r2 values larger than 0.7 for 67% of reservoirs used in the validation study, indicating that the surface water area can be used as a proxy of storage in many situations when analyzing reservoir storage dynamics.

**Figure 1:**
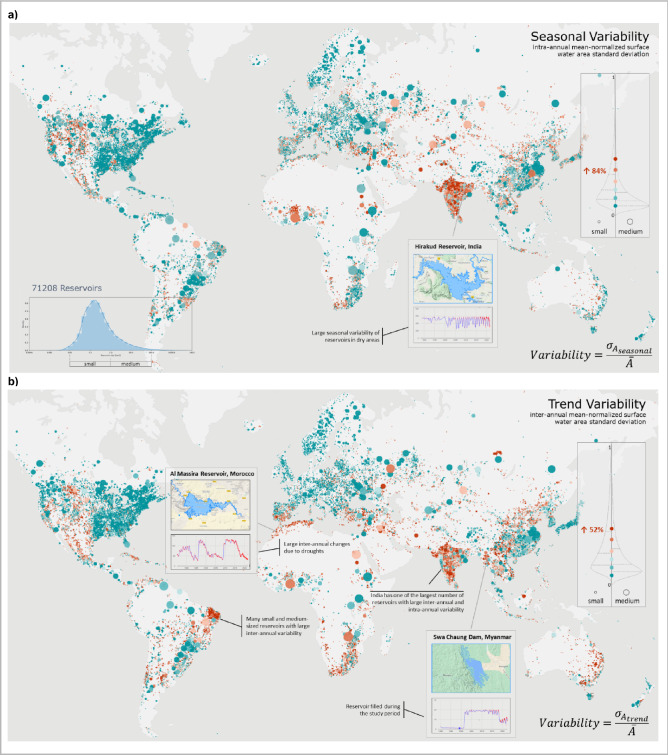
Overview of the reservoirs included in the database and their surface water area dynamics. The mean-normalized intra-annual (seasonal) (**a**) and inter-annual (trend) (**b**) variability of the surface water area of reservoirs, computed as a standard deviation of trend/seasonal components of surface water area time series divided by its mean area. Seasonal/Trend decomposition of surface water area time series is performed using *statsmodels* Python library^32^. The map is generated using Unfolded Studio^33^. Map: https://studio.unfolded.ai/public/189a4711-8fd7-4884-ac49-d97e9f93796e.

Besides the global statistics on the surface water area of reservoirs in the last 36 years (1985–2021), we also studied their intra-annual and inter-annual variability. Figure 1 shows how the surface water area of small and medium-sized reservoirs varies at inter-annual (year-to-year trend) and intra-annual (seasonal) scales. Note that 479 reservoirs with a mean area larger than 100 km^2^ are also depicted in the maps but were excluded when computing trend/seasonal variability due to lower accuracy. We did not analyze the origin of inter-annual trend variability, which could be caused by reservoirs being filled during the study period, decommissioned, or large climate variabilities.

We also investigated if the seasonal/trend variability of surface water area varies significantly for different geographical regions with the established dataset. We did not find any significant spatial relations, implying that the found relationship between variability and size of surface water bodies, when investigated over a large enough set of samples, applies globally, regardless of the climate region or landscape.

In the remainder of this paper, we investigate three possible use cases for our reservoir surface water area dynamics database, assuming sufficient satellite data is available in near real-time.

### Claims regarding water scarcity

Climate change increases the frequency and severity of extreme weather events, such as floods and droughts. But politicians may be using this fact to hide some of the real culprits behind water scarcity, such as increasing water withdrawals and poor water resources management. Objective and timely information on surface water resources can support or counter claims regarding water scarcity and/or its principal drivers. We demonstrate this by analyzing surface water dynamics in Turkey in the years leading up to January 2021.

**Figure 2:**
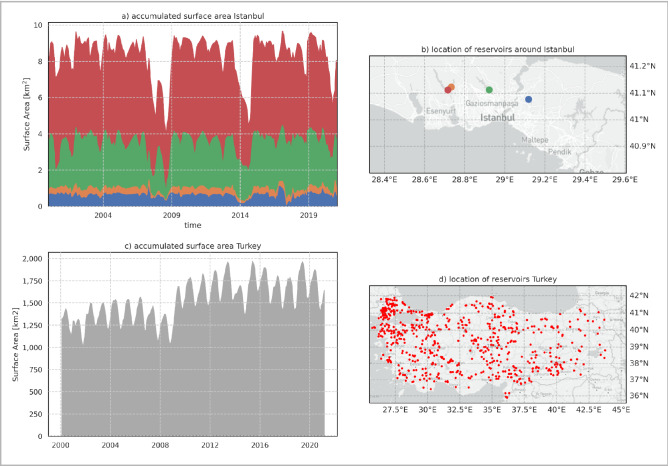
Surface area time series for reservoirs in Turkey. (**a**) Accumulated time series of surface area for the four reservoirs neighboring Istanbul; (**b**) Location of reservoirs, plotted in subplot (**a**). The colors are commensurate with the time series plots. (**c**) Time series of total water surface area from 2000 until 2020. (**d**) Location of water bodies, of which surface area was included in subplot (**c**). The figure is generated using Matplotlib^34^ and Cartopy^35^ python libraries.

In mid-January 2021, The Guardian reported that water availability was critically low across Turkey as it faced “*the most severe drought in a decade”*^33^. In particular, Istanbul was reported to be hit hardest, with reservoir levels receding, such that curtailments were needed. Focussing on Istanbul first, Fig. 2a,b show the surface area time series for the largest reservoirs around Istanbul. The results indeed confirm this and reveal that these reservoirs were indeed at a low level, but also, that more dramatic drought conditions have occurred in the past, particularly a two-year drought in 2007–2008 and another in 2014. It should be noted that Istanbul’s population has grown dramatically over the past decades, making this region a severely water-stressed one due to rapidly increasing demand and, therefore, highly vulnerable to the declining rainfall across the Mediterranean as a result of climate change^34^.

We also analyzed the accumulated surface water of all reservoirs in our database over the entire country to support the findings reported by The Guardian article. Figure 2c,d show the behavior of the accumulated surface water area (c) in time, with the locations of the reservoirs (d) considered. Our analysis for Turkey only includes reservoirs larger than an average surface water size of 10 hectares and smaller than 50 km^2^. The graph does not reveal the drought as significantly as the results for Istanbul. It does, however, demonstrate that there is a clear upward trend in the long-term surface water. After further inspection, we found that this results from the development of entirely new surface water bodies. We visually inspected several locations where new reservoirs were constructed using the Aqua Monitor tool^17^ and confirmed that their surface water area time series indeed increased significantly from almost zero. The significant change in surface water over the year 2009 is partly due to the refilling of existing water bodies after the drought of 2008 and partly due to the filling of entirely new impoundments, such as Manyas, Boyabat, Çekerek, and a number of others.

Although surface water area estimates do not provide the means to monitor per-capita available water resources, the above evidence demonstrates that surface water, and volumetric water resources availability (because of the interrelation between surface area and volume), was much higher than it was during the 2008 drought. This was partly due to the construction of new dams and resulting impoundments over a short amount of time.

The above example demonstrates that surface water monitoring may assist in answering attribution questions around water scarcity. It also indicates that essential contextual information is needed to understand the attribution problem, such as population growth numbers and translation of available surface water resources into relative units such as per-capita available water resources. Also, the ability to distinguish water resources from new impoundments will assist in understanding the development of surface water availability in a given country against the challenges imposed by drivers of scarcity, something essential to monitor progress in Sustainable Development Goal 6. It is then vital to make users of the information understand the complexity of these compounding effects on water scarcity in a digestible manner. It is also important to note that we focus only on surface water resources without looking at other sources such as groundwater.

### Drought early warning, response, and food insecurity

Another logical use case for near real-time monitoring of reservoirs is drought early warning and water and food insecurity early warning. Suppose multiple reservoirs in multiple basins or countries suffer from very low surface water amounts compared to the normal situation. Detecting this early may be an early warning signal for degrading food production and thus potentially upcoming food insecurity. If this situation is observed in an area that strongly relies on local food production at the onset of the dry season, then this may imply food shortages in the forthcoming season. Early observations may then be used to organize and implement water use limitations, redistribution towards priority uses (such as drinking water supply), government food supply and early aid mobilization by the international disaster relief and development communities to relieve water and food insecurity.

Here we will use recent droughts in South Africa, including the well-documented “Day-Zero” drought impacting Cape Town in 2017^36,37^, to demonstrate this, and the earlier, but much larger-scale drought, impacting the entire region in 2015/2016, demonstrating the importance of large-scale monitoring for water rationing decisions or pre-allocation of food resources.

During the severe drought that affected Cape Town in 2017, concerns about the drought were so severe that plans were made to shut down all but essential services^38^. But while this drought was very well captured on storage time series and reported on in the media, the large-scale drought of 2015/2016, which affected water resources and food availability in the entire region, including the Limpopo, Incomati, Umbeluzi and Zambezi basins, has never been adequately described in terms of storage anomalies . Most studies have focused on investigating the climate phenomena leading to the drought^39^, rainfall and runoff anomalies, or the response of institutions to the drought^40^. However, surface water storage has not been monitored at a large scale, althoughthis variable may serve as a good indicator for the state of water resources^41^, also because it integrates the history of rainfall, runoff, and water use over time, including possible multi-annual storage effects.

**Figure 3:**
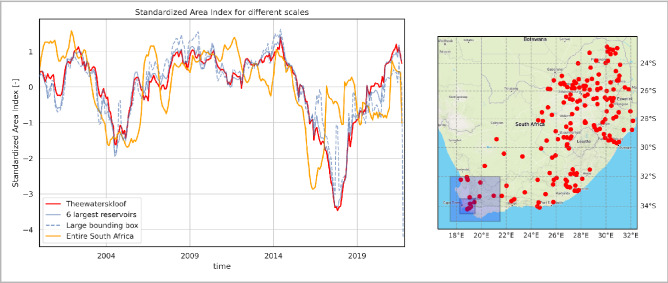
Water dynamics in South African reservoirs. Left: Standardized Area Index (SAI) for Theewaterskloof dam, the six largest dams (> 1 km^2^) in our database around Cape Town (dark blue bounding box, right side), and a larger bounding box (light blue box, right side). Right: Map in Red all reservoirs in our database > 1 km^2^ in South Africa. All of these are included in the SAI of “Entire South Africa” in the left graph. The period 2000 until 2020 is used as reference climatology. The figure is generated using Matplotlib^34^ and Cartopy^35^ python libraries.

We describe the surface water conditions in a way similar to the Standardized Storage Index (SSI)^42^. SSI shows how much the current storage deviates from normal conditions in the given month, measured in standard deviations.We introduce a Standardized Area Index (SAI) in the same way as SSI, looking atthe variability of surface water area of reservoirs over larger areas. This allows for an equivalent comparison between all reservoirs in a given area, regardless of their size, seasonal control that may be applied, or storage to runoff ratio as a measure of multi-year storage capacity. Figure 3 shows the SAI applied to surface water area time series (instead of storage) for respectively: Theewaterskloof Dam, the largest dam providing water to Cape Town; the six largest reservoirs (by surface area) around Cape Town in our database, and all water surfaces together of impoundments larger than 1 km2 across entire South Africa. The results show that by November 2017, the storage anomaly in Theewaterskloof Dam was more than three standard deviations under normal expected conditions, proving that this drought was the worst recorded in the last thirty-six years. This also holds for the entire surroundings of Cape Town. Interestingly for South Africa, on average, this same period was quite normal (orange line). For the whole country, the earlier occurring 2016 drought comes up very clearly.

**Figure 4:**
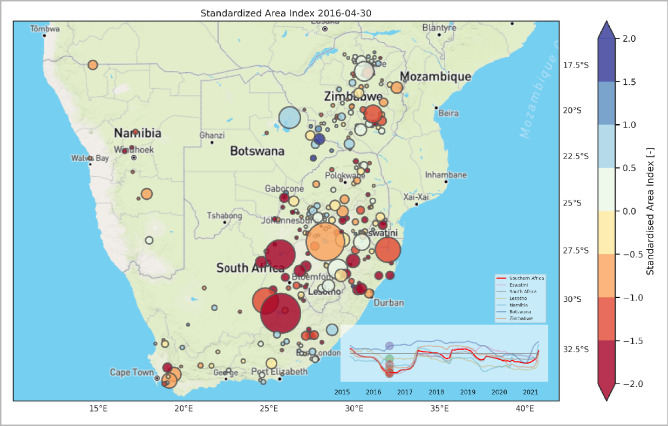
Standardized Area Index for all reservoirs > 1 km^2^ in South Africa, Eswatini, Lesotho, Namibia, Botswana, and Zimbabwe in April 2016. The size of the circles represents the mean surface water area of the reservoirs during 1985–2021. The colors of the time series (lines), and the plotted moment in time (circles) in the inset plot, correspond with the country in the legend. The figure is generated using Matplotlib^34^ and Cartopy^35^ python libraries.

To visualize the 2016 drought geospatially, Fig. 4 shows how the drought manifested itself over reservoirs in the entire region, including the surroundings of South Africa, notably Eswatini, Lesotho, Namibia, Botswana, and Zimbabwe. We show here the conditions at the beginning of the dry season of 2016 (April), a moment at which the information could be used as an early warning signal. It demonstrates how severe conditions such as “Day-zero” may be monitored at a much broader scale and earlier time, offering clear early warning signals for water and food shortages and possibilities for pre-allocation of drought response resources at the national but possibly also at transboundary level. Understanding the effects of drought at the transboundary basin level, where drought affects also the other riparian states may help foster cooperation instead of triggering conflicts.

### Transboundary water conflicts and cooperation

Monitoring water resources use in small and medium-size reservoirs can be a considerable contributor to conflict prevention and mitigation in internationally shared basins. A significant share of all conflicts between riparian states emerges in relation to water allocation and/or the development of infrastructure for irrigation and hydropower generation^43^, especially if states favor unilateral strategies for doing so. Very often, such efforts spark disagreement or full-fledged conflict as other riparian states get concerned about the (potential) negative environmental and socioeconomic effects of such projects on their own water resources use opportunities and a potential violation of the principle of no significant harm, which is considered a cornerstone of cooperation over shared water resources^43,44^. As states accord strategic or even security relevance to water resources or consider dams as emblematic symbols of nation-building^45^, dams and their reservoirs are tied to a political conflict potential that often exceeds the actual water resources use dimension of those. De-politicizing or de-securitizing the dams discourse and instead engaging in creating a joint understanding of water resources use and possible change across the entire basin can thus be an important factor in mitigating conflict and fostering cooperation.

The countries in the Tigris-Euphrates basin (Turkey, Syria, Iraq, and Iran) have unilaterally developed irrigation and hydropower schemes over the last 50 years, intensifying water use in a water-scarce basin. This led to an intensification of tensions over shared water resources in a region that also suffers greatly from several prolonged violent conflicts beyond the water sector. The potential of monitoring basin-wide water resources may support future steps towards cooperation as it levels the information playing field between all riparian states.

Disputes concern, in addition to the protracted dispute between Turkey and Iraq, between Iran and Iraq. Iran is storing water from tributaries to the Tigris (such as the Sirwan and the Little Zab rivers, together accounting for about 25% of the Tigris’ annual flow) flowing into Iraq, and diverting water eastwards to ease the severe water scarcity large parts of the country are facing and the socioeconomic as well as political implications that come with it^46^. In order to do so, Iran has built more than 600 dams across the entire country in the last decades, some of those on rivers flowing to Iraq. This is done without sharing data with neighboring Iraq, not least because cooperation in general and data and information exchange in particular between both states are limited^46^. Individual water crises in two riparian states are thus aggravated by unilateral measures to alleviate such crises and have led to tensions between the two countries.

Access to data on reservoir water dynamics and thus also reservoir operation could provide a basis for both states to engage in cooperative approaches to using the scarce water resources they share in more efficient ways. While political willingness to share data remains low in the region, open-access data that does not formally originate from one country (and can therefore be rejected or questioned by the other country) could—albeit most likely not perceived with open arms—pave the way towards more leveled exchange and a shift from accusations based on assumption to negotiations based on neutral and externally provided facts.

**Figure 5:**
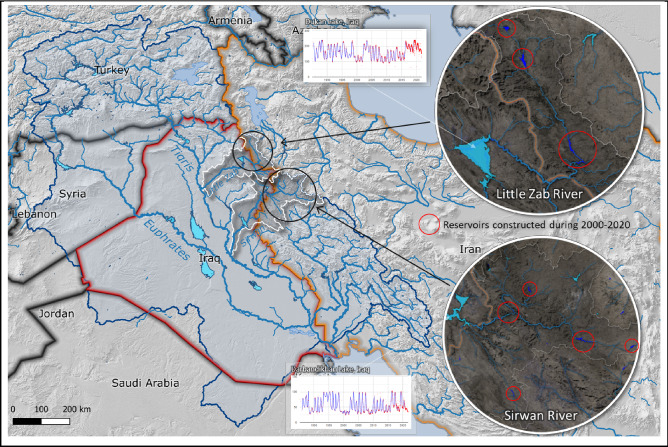
Reservoirs in the transboundary basin of Tigris–Euphrates and small reservoirs constructed during 2000–2020 in Iran in the upstream part of the Sirwan and the Little Zab rivers. The figure is generated using QGIS 3.18^47^ and QGIS Google Earth Engine plugin^48^.

Figure 5 shows the transboundary basins of the Little Zab and Sirwan rivers as well as the main reservoirs located in this area. While our database contains surface water dynamics of the two large reservoirs on the Iraq side (Dukan and Darbandikhan Lakes), a number of smaller reservoirs constructed upstream during 2000–2020 (indicated in red) were mapped by making use of the Aqua Monitor algorithm^49^.

## Discussion

The advent of cloud computing resources and decentralized data storage makes it possible to establish worldwide monitoring services. This provides new scientific insights into how water bodies behave in time, as shown by several previous authors. The implications for science and society may be vast. Such monitoring capabilities, especially when extended from surface areas to water volumes, and complemented by socio-economic data, will also support the monitoring of water-related global targets, such as those related to the Sustainable Development Goals. In this paper, we showed that in particular small water bodies (< 100 ha) show a large inter- and intra-annual variability in available water resources, alluding to implications for the security of food production, especially for societies that rely on smaller water bodies. Monitoring the trends in reservoir numbers (i.e., new reservoirs appearing), their available resources, how variable these resources are overtime, and possible changes in both may provide the baseline for such monitoring. Furthermore, the behavior of new and existing reservoirs may open debate on their impact on society and the environment and how such reservoirs could be better managed in order to protect both. Moreover, in transboundary basins, such information can also support conflict mitigation and better water management across riparian states. Ideally, they may even result in more efficient multipurpose use of reservoirs in multi-reservoir basin systems. To be able to truly understand impacts and trigger decision making to improve water management, more insights are required—for instance which ecosystems may be threatened by them, which communities are served by which water body, and how reliant such communities are on surface water alone. Such information remains highly localized and will require local data collection efforts in conjunction with satellite data analyses. Combining global water resources observations with more localized socio-economic details is key to understanding the exact impacts on communities. It will make our near real-time estimates much more helpful for many possible end-users.

The case studies demonstrate convincing examples where global scale operational reservoir monitoring at detailed spatial (< 100 m) and temporal (weekly) scales have two important implications: first, any stakeholder (not only the one operating or controlling the water body) can monitor these in real-time. This may result in a level playing field on water information during politically and socially sensitive negotiations about water entitlements, transboundary agreements, and a better basis for law enforcement provided that our observations are formally recognized. Second, we foresee that near real-time observations can be a significant contributor to assisting water management in light of current and future water crises, helping to reduce the vulnerability of people and societies to scarcity^41,50^. Real-time observations, provided they are timely and skillful enough, may inform e.g., improved real-time water accounting, improved dam operations and curtailing decisions, or early mobilization of humanitarian aid. They will form an essential input for seasonal water resources forecasting, given that the present-day state of water resources provides skill for such forecasts in the first weeks to months ahead^41,51,52^.

The prospects are that governments at local as well as national levels, international organizations, humanitarian aid agencies and NGOs can be provided with better and more localized information on water issues (too much or too little) and act upon those earlier; the reinsurance industry may tailor their payouts and premiums based upon monitored water shortages, and energy utilities are enabled to monitor hydropower potential for the coming months based on (upstream) reservoir states and potentially adjust power production based on anticipated rainfall and water availability and changing energy demands in a shared regional market to give a few examples.

### Limitations and future developments

Our current analysis is limited to the impoundments’ surface area alone. Our dataset can be used to indicate storage variability or % of reservoir filling, assuming that the reservoir banks are not too steep to ensure that water level changes result in surface water area changes. For many applications, however, volumetric time series are a must. For instance, the Food and Agricultural Organization evaluates water accounting for many countries, requiring a month-to-month understanding of cubic-meter storage of resources (besides the month-to-month fluxes). The surface area variations alone are not sufficient. Moreover, our surface area dynamics dataset will not offer enough information where reservoir banks are steep, resulting in a small variability of the water surface area. The logical next step would be to combine our dataset with altimetry for both cases. Cooley et al.^27^ already show the vast amount of possible observations that can be made with the ICESat-2 satellite for both small and large reservoirs. Busker et al.^25^ computed surface volume time series for 137 large water bodies, using a combination of monthly surface area estimates^18^ and the DAHITI water level archive^53^. The new SWOT mission^54^ will offer an opportunity to establish storage-area or storage-depth relationships, which can then be used for real-time monitoring in an operational setting. In addition, a large additional value may lie in the adding of dam properties and installations to estimate live storage, potential hydropower production, and the likelihood of spills and spillage amounts.

Further, the provided coverage in this paper is nowhere near complete, and limited curation has been done. In particular, many small reservoirs are missing, the maximum extent of reservoirs is not always correct in existing vector maps, and the prior water occurrence probability, used to fill gaps in unobserved parts of the water body for cloudy images, can be significantly improved. One of the possible extensions of our dataset could be to attribute large inter-annual changes of reservoirs by analyzing surface water area time series, identifying newly constructed reservoirs, periods of drought, or decommissioned reservoirs. Further extension of our database, curation of the reservoirs’ existence, extent, and classification in natural versus man-made remain for future work.While our algorithm demonstrates excellent results for arid/semi-arid environments, several challenges remain open. Harmonized satellite imagery from Landsat and Sentinel optical missions results in high-frequency observations in reservoirs in these areas but provides limited results in regions with significant cloud cover presence. A solution would be to extend the monitoring with the free data from the Copernicus Sentinel-1 SAR mission satellites. The use of a simple spectral index approach, even with the use of dynamic thresholding, makes it challenging to discriminate water from snow and ice or hill shadows, including more spectral information and/or auxiliary datasets (such as height above the nearest drainage), are expected to improve the algorithm accuracy in these cases. Furthermore, either multi-class Otsu and/or the use of more advanced machine learning methods, such as Deep Neural Networks, may further improve the algorithm's applicability.

## Methods

Our method to derive water dynamics of reservoirs builds upon previous studies focusing on the mapping of dams and water bodies^30,31,55–57^. We derive surface water area dynamics of reservoirs by analyzing freely available medium-resolution satellite images acquired in the last 35 years by NASA's Landsat and ESA’s Copernicus Sentinel missions. Our water detection algorithm was applied individually for all 71,208 water bodies and every satellite image intersecting with the given water body. The method is implemented using the Google Earth Engine platform to process satellite imagery, which perfectly suits the need to process a multi-petabyte satellite dataset. Still, the overall generation of the dataset took about six months of run time.

One of the first challenges we had to overcome was that no harmonized global water reservoirs dataset exists today that maps both small-, medium-, and large-sized reservoirs. We have combined vector maps of water bodies from multiple vector datasets and attributed them as artificial water reservoirs if they intersect with dams (or are located close to dams) or are already attributed to reservoirs originally. Dams used here were collected from multiple existing datasets^29,30,54,55^. This harmonized reservoir vector dataset was then used to derive surface water dynamics from satellite images.

Accurate detection of surface water from optical satellite imagery implies solving several challenges. Firstly, the water/land boundary can be fully or partially occluded by clouds or shadows from clouds or hills. Secondly, spectral properties of the land and water surface near the land/water boundary may vary significantly. Also, multiple effects can be present in larger reservoirs resulting in additional noise, such as water slope variability (due to wind or water flow) or the presence of vegetation or other masses floating on the water surface (ice, aquaculture). These challenges make using naive water detection methods less feasible for accurately estimating the surface water area of water bodies. In the sections below, we further describe how we tackled these challenges.

**Figure 6:**
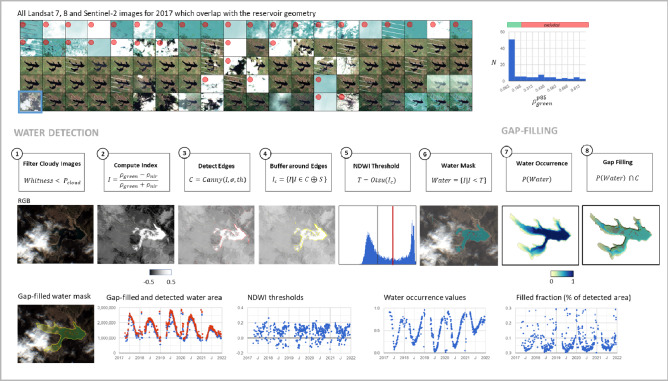
Method of surface water area detection for reservoirs. The algorithm to compute surface water consists of the next steps: (1) Select satellite images least cloudy over the reservoir area (2) Compute spectral water index (NDWI here, but could be any spectral index suitable for surface water detection) (3) Apply Canny edge filter to detect water/land edges (additional steps can include edge suppression based on the spectral properties around edges) (4) Define sampling region for pixels surrounding water/land edges (5) Sample spectral index values within the buffer computed during step 4 and compute optimal threshold using Otsu method used to (6) Compute the surface water area (7) Select surface water occurrence (8) Fill gaps (false-negatives) in the resulting water mask, remove incorrectly detected water (false-positives) by sampling water occurrence along water edges, and compute the final filled surface water area mask by clipping water occurrence at a given probability and combining it with the detected water mask. The figure was generated using Google Earth Engine Code Editor tool^14^. Code: https://code.earthengine.google.com/54602c8309f44cf83e42cf93a854dc49.

Numerous algorithms and datasets have been developed over the last decade focusing on the accurate estimation of surface water dynamics from optical satellite imagery^18,58–63^. A key requirement to use those algorithms that can classify water where it is occluded due to clouds^24,64,65^. To detect water mask in every satellite image that intersects with a given reservoir geometry, we first discriminate surface water using NDWI spectral index by applying the method of local thresholding based on the Canny edge detector and a binary version of the Otsu thresholding algorithm^61,66^. The water detection step is followed by the gap-filling step, eliminating false-negative (i.e., water pixels detected as non-water) water detection. We did not use water occurrence to remove false-positive pixels (i.e., non-water pixels classified as water) due to the low accuracy of the water occurrence dataset. Instead, we only excluded pixels detected as water where NDWI values are less than -0.15, which corresponds to land in most cases. During the gap-filling step, we combine detected water masks with the water occurrence dataset^18^ to determine which areas belong to false negatives and combine them with the detected water mask to obtain the gap-filled water mask. Finally, the resulting surface water area time series are post-processed with a temporal outlier filtering (quantile-based) to remove the remaining errors. Our algorithm does not explicitly apply cloud masking for performance reasons; instead, we filter out bright images fully covered by clouds using the global cloud frequency dataset^67^. For every satellite image overlapping with a reservoir, we compute the top-of-the-atmosphere (TOA) reflectance value which corresponds to an 85% quantile over the maximum reservoir area (regional reducer). This metric is then used to indicate the whiteness of every image, with high values corresponding to images fully covered by clouds. Combined with the average annual cloud brightness observed on average over the reservoir area, we filter out the cloudiest images. Cloud pixels present in the remaining images are corrected during the post-processing step. The outline of this multi-step algorithm is shown in Fig. 6.

### Validation

**Figure 7:**
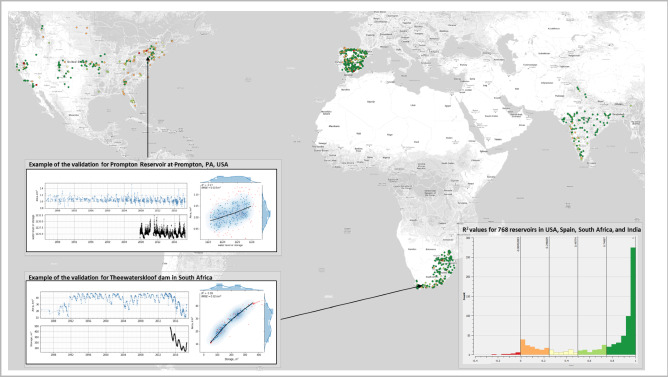
Results of the validation of the reservoir surface water area dataset using 786 in-situ time series of water level and storage for the USA, Spain, South Africa, and India. Frequency histogram chart (bottom-right) shows the distribution of the coefficient of determination (R^2^) for all reservoirs used in the validation; charts at the left show examples of the performed validation procedure, with a good fit (bottom, R^2^ = 0.99, RMSE = 0.92 km^2^) for the Theewaterskloof reservoir in South Africa, Mediterranean climate zone and less good (top, R^2^ = 0.27, RMSE = 0.03 km^2^) for the small Prompton Reservoir in the USA located in humid continental climate zone. The figure was generated using Google Earth Engine Code Editor^14^ and the charts were generated with Matplotlib^34^ and Seaborn^68^ libraries. Code: https://code.earthengine.google.com/29230bfe2978625f0981890b1565c6b6.

To validate the algorithm’s performance, we compare established surface area time series against in-situ measurements of water levels or storages with an observation frequency of daily or higher for 768 reservoirs in Spain, India, South Africa, and the United States. Figure 7. Shows an overview of validation locations used and the distribution of correlation coefficients computed per validated reservoir. The figure also shows an example of the relationship between our surface area estimates and in-situ storage time series for the Theewaterskloof dam in South Africa. Our time series correlate well with in-situ observations, with r2 values higher than 0.7 for 67% of reservoirs used in the validation. Performance degrades for reservoirs where surface water area variability is small (temperate climate zone or steep reservoir banks) or where in-situ measurements are of lower quality. An example of such a case is also provided for the Prompton Reservoir (United States). To monitor water storage changes in these reservoirs, alternative methods to monitor its variability are needed such as altimetry (in case storage changes more with surface elevation) or the addition of synthetic aperture radar (SAR) satellite imagery (in case hardly any cloud-free imagery is available). Detailed results of the validation study and datasets used during the validation are included as supplementary materials.

The method used during the validation includes a temporal interpolation of daily in-situ measurements for every satellite-derived measurement point. The next step involves the removal of outliers using thresholding of the 2d kernel density estimation applied to the scatter plot^69^. The final step computes R^2^ and RMSE after applying a non-parametric regression using the LOWESS algorithm^70^ using the *statsmodels* python package^32^.

## Supplementary Information

Supplementary Information.

## Data Availability

All new data and code generated in this research are available under the terms of Creative Commons BY 4.0 (for the data) and Apache 2.0 (for the code) licenses. Datasets and supplementary materials generated during this study are accessible from the supplementary materials document below. The source code used to produce datasets is accessible from: https://github.com/global-water-watch/research-reservoir-water-dynamics. For more information about this research and to access the demo app visit: https://globalwaterwatch.earth.
