# Author Correction: High-resolution surface water dynamics in Earth’s small and medium-sized reservoirs

**DOI:** 10.1038/s41598-022-20467-2

**Published:** 2022-09-20

**Authors:** Gennadii Donchyts, Hessel Winsemius, Fedor Baart, Ruben Dahm, Jaap Schellekens, Noel Gorelick, Charles Iceland, Susanne Schmeier

**Affiliations:** 1 Deltares, Delft, The Netherlands; 2 Planet Labs PBC, Haarlem, The Netherlands; 3 Google, Zürich, Switzerland; 4 World Resources Institute, Washington, DC USA; 5 IHE Delft, Delft, The Netherlands; 6 Delft University of Technology, Delft, The Netherlands

Correction to: *Scientific Reports* 10.1038/s41598-022-17074-6, published online 12 August 2022

The original version of this Article contained an error in the Data Availability section where

“All new data and code generated in this research are available under the terms of Creative Commons BY 4.0 (for the data) and Apache 2.0 (for the code) licenses. Datasets and supplementary materials generated during this study are accessible from the TODO: upload and add Zenodo link here. The source code used to produce datasets is accessible from: https://github.com/global-water-watch/research-reservoir-water-dynamics. For more information about this research and to access the demo app visit: https://globalwaterwatch.earth.”

now reads:

“All new data and code generated in this research are available under the terms of Creative Commons BY 4.0 (for the data) and Apache 2.0 (for the code) licenses. Datasets and supplementary materials generated during this study are accessible from the supplementary materials document below. The source code used to produce datasets is accessible from: https://github.com/global-water-watch/research-reservoir-water-dynamics. For more information about this research and to access the demo app visit: https://globalwaterwatch.earth.”

The original Article has been corrected.

