# Supplementary material for: High-resolution surface water dynamics in Earth’s small and medium-sized reservoirs
Source: Sci Rep. 2022 Aug 12;12:13776. doi: 10.1038/s41598-022-17074-6 (PMC9374738; doi:10.1038/s41598-022-17074-6)
Supplement: Supplementary file 1 — Supplementary Information. [file 41598_2022_17074_MOESM1_ESM.docx]

**Supplementary materials** for the High-resolution surface water dynamics in Earth’s small and medium-sized reservoirs

Gennadii Donchyts^1,6,*^, Hessel Winsemius^1,6^, Fedor Baart^1,6^, Ruben Dahm^1^, Jaap Schellekens^2^, Noel Gorelick^3^, Charles Iceland^4^, Susanne Schmeier^5^

^1^Deltares, Delft, The Netherlands

^2^Planet Labs PBC, Haarlem, The Netherlands

^3^Google, Zürich, Switzerland

^4^World Resources Institute, Washington D.C., USA

^5^IHE Delft, Delft, The Netherlands

^6^Delft University of Technology, Delft, The Netherlands

*gennadii.donchyts@deltares.nl

This document describes supplementary materials for the **High-resolution surface water dynamics in Earth’s small and medium-sized reservoirs** research paper in Nature Scientific Reports. This includes the dataset containing time series of surface water area for 71208 reservoirs globally derived from optical Landsat and Sentinel-2 satellite imagery acquired during Jan 1985 ... Sep 2021 as well as the validation dataset and the source code used to produce these datasets.

The data files listed below can be downloaded from the following data repository: <https://doi.org/10.6084/m9.figshare.20359860>

See also the following GitHub repository for the source code of the algorithms and links to the app to explore the dataset: <https://github.com/global-water-watch/research-reservoir-water-dynamics>

**Table 1** Files included in supplementary materials and a short description

| **src.zip/** |  |  |
| --- | --- | --- |
| code-js/global-water-watch-paper-area/  app-gww-area-prototype.js  app-gww-area-validation.js  figure1.0-reservoirs-styled.js  figure6.0.js  figure6.1-gallery.js  figure7.0-validation-histogram.js  surface-water-area-algorithm.js  surface-water-area-algorithm-test.js | JavaScript version of the algorithm, prototype app, and source code for some of the paper figures, can be also accessed using Google Earth Engine: <https://code.earthengine.google.com/?accept_repo=users/gena/global-water-watch-paper-area> |  |
| code-py/ee-packages-py/  eepackages/  applications/  waterbody_area.py | Python package containing a set of Earth Engine utilities (also available in https://github.com/gee-community/ee-packages-py and as a PyPi package) |  |
| notebooks/ | Jupyter Notebook source code of the post-processing steps used to generate derived time-series, interactive version of the above scripts |  |
| 01-daily-to-monthly.ipynb |  |  |
| 02-monthly-to-aggregated-COUNTRY- BASIN.ipynb |  |  |
| 03-monthly-to-aggregated-GADM.ipynb |  |  |
| 04-generate-variances.ipynb |  |  |
| script/ | Scripts used to: |  |
| 01_update_reservoir_data.py | batch-generate the dataset |  |
| 02_merge_time_series.py | merge the newly generated time series |  |
| 03_daily_to_monthly.py | generate monthly time series |  |
| 04_aggregate_monthly_COUNTRY_BASIN.py | aggregate time series by country and basin |  |
| 05_aggregate_monthly_GADM.py | Aggregate time series by administrative levels |  |
| **shp.zip/** |  |  |
| reservoirs-locations-v1.0.dbf  reservoirs-locations-v1.0.prj  reservoirs-locations-v1.0.shp  reservoirs-locations-v1.0.shx | Reservoir locations and statistics based on area time series (mean, median, sd_intra, sd_inter, rsd_intra, rsd_inter)  sd_intra, sd_inter are computed for different time series components after seasonal-trend decomposition (STL)  rsd_intra, rsd_inter are computed as relative ratio of changes as rsd_intra = sd_intra / mean, rsd_inter = sd_inter / mean |  |
| reservoirs-v1.0.dbf  reservoirs-v1.0.prj  reservoirs-v1.0.shp  reservoirs-v1.0.shx | Maximum (buffered) extent geometry of reservoirs used to extract surface water area time series  reservoir geometry was identified from multiple databases such as OpenStreetMap, HydroLAKES, and many dam dataset |  |
| **time_series_area_raw.zip/**  *.csv | Time series derived from EO data, raw time series with all metainformation (satellite mission, quality metrics, etc.), file format: CSV |  |
| **time_series_area.zip/**  *.csv | Time series of surface water area (only time and area files), derived from the raw time series |  |
| **time_series_area_monthly.zip/**  *.csv | time series of surface water area aggregated to monthly time steps |  |
| **time_series_area_by_gadm0.zip/**  *.csv | time series of total surface water area per administrative level using GADM polygons level 0 | |
| fid_gadm0_mapping.csv | mapping of reservoir fid to GADM GID_0, multiple GADM areas are possible per reservoir |  |
| **time_series_area_by_gadm1.zip/**  *.csv | time series of total surface water area per administrative level using GADM polygons level 1 |  |
| fid_gadm1_mapping.csv | mapping of reservoir fid to GADM GID_1, multiple GADM areas are possible per reservoir |  |
| **time_series_area_by_gadm2.zip/**  *.csv | time series of total surface water area per administrative level using GADM polygons level 2 |  |
| fid_gadm2_mapping.csv | mapping of reservoir fid to GADM GID_2, multiple GADM areas are possible per reservoir | |
| **validation.zip/** |  |  |
| figures/ | validation figures comparing in-situ measurements or estimates (water levels, storage) vs EO-based surface water area estimates  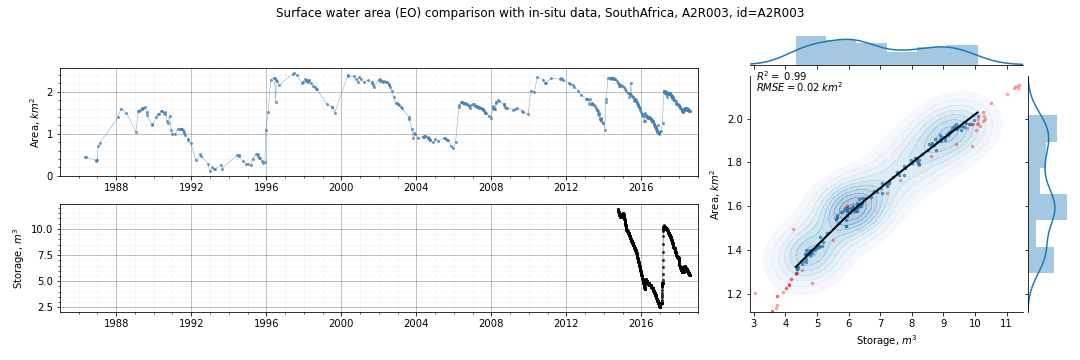 |  |
| shp/ | locations of validation points where in-situ data were collected per country (India, USA, South Africa, and Spain) |  |
| time_series/ | Validation time series, including in-situ measurements of water level and storage and EO-based surface water area |  |
| map.png | an overview map showing the goodness of fit for validation points (r2 colored from red to green)  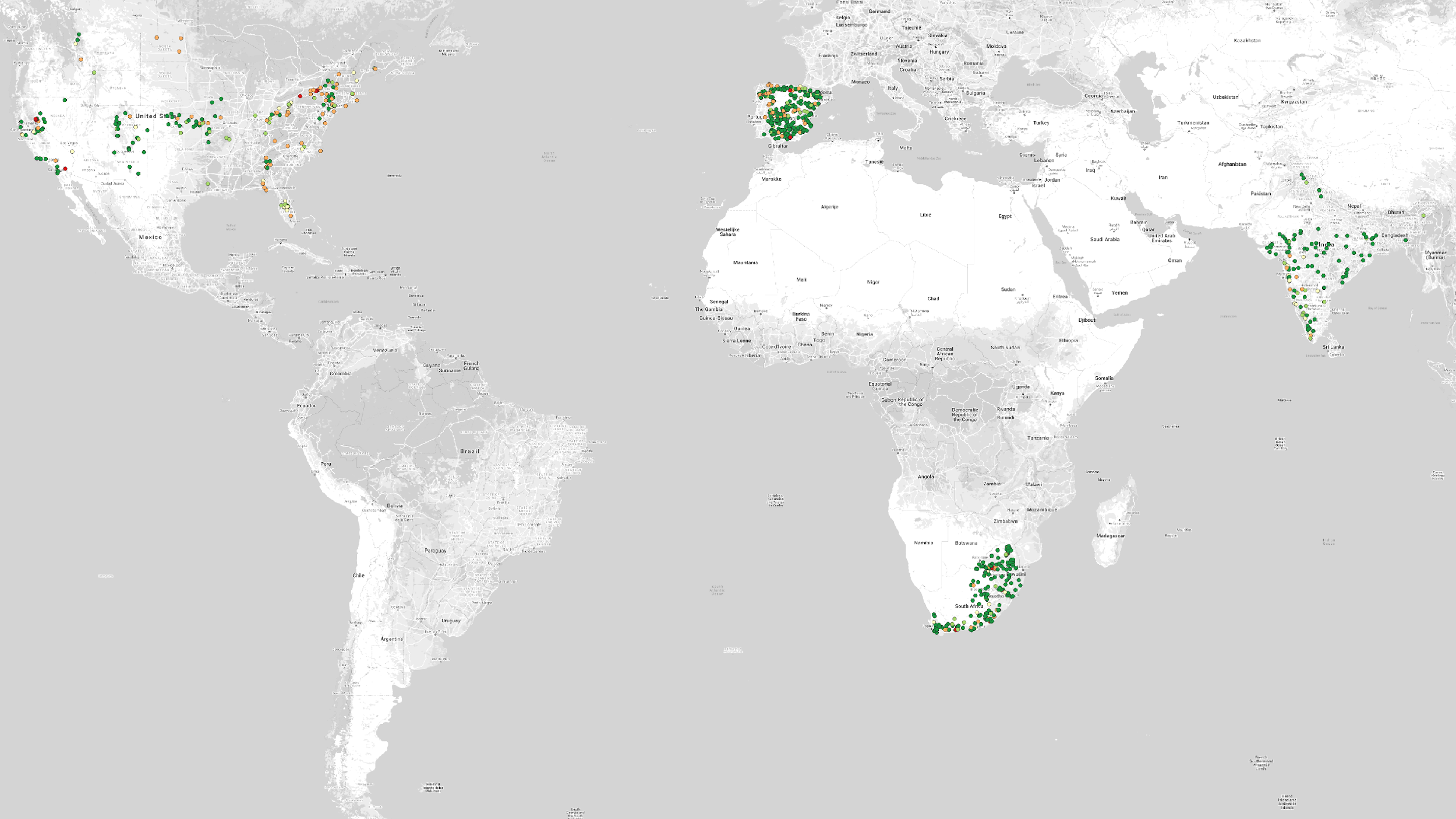 |  |
|  |  |  |

Filename conventions for CSV files generated per reservoir:

<fid>.csv, where <fid> is a unique reservoir id defined in the reservoirs-locations-v1.0 and reservoirs-v1.0 shapefile.

<GID_0>.csv is used for time series file names aggregated by GADM level 0

<GID_1>.csv is used for time series file names aggregated by GADM level 1

<GID_2>.csv is used for time series file names aggregated by GADM level 2

<COUNTRY_ISO>.csv is used for time series file names aggregated by country

<BASIN_ID>.csv is used for time series file names aggregated by hydrological basin (according to FAO basins.

**One the new reservoirs constructed in Turkey between 2002 and 2012**

In the current study, we did not map newly constructed reservoirs and only included reservoirs already mapped by other vector datasets. However, to confirm our hypothesis that the construction of new reservoirs caused the increase in surface water area in Turkey around 2010, we have visually inspected surface water changes using the Aqua Monitor tool (http://aqua-monitor.deltares.nl). We could confirm that the surface water area of these reservoirs has increased from almost zero during that period, as indicated in Fig 1.

| **Figure 1. Surface water changes (transition from land to water and from water to land) between 2002 and 2012 years revealing new reservoirs constructed between 2002 and 2012**  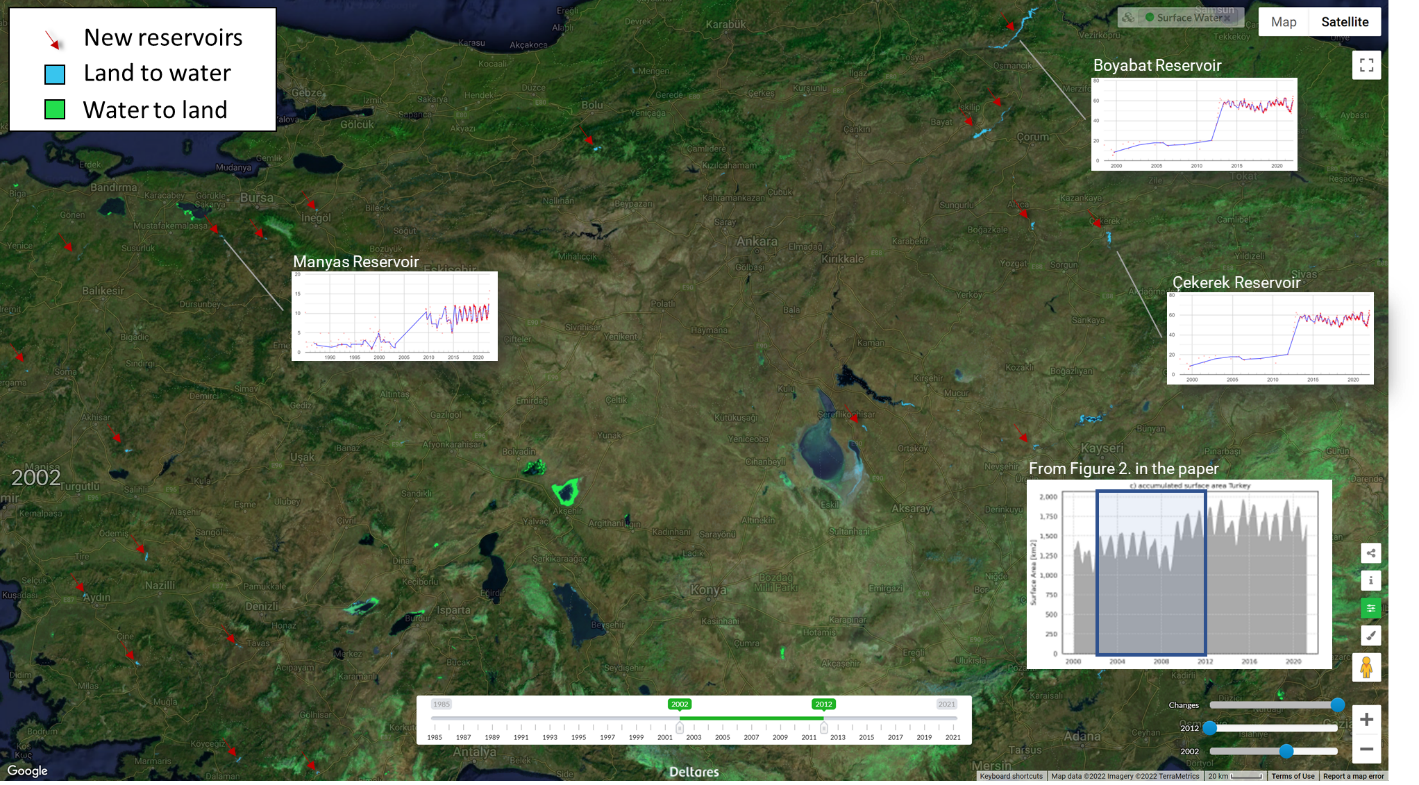  Link: <https://aqua-monitor.appspot.com/?mode=dynamic&from=2002&to=2012&view=38.670,31.448,8z> |
| --- |

**On the comparison of surface water area time series in-situ datasets and GRACE**

We compared our dataset with daily in-situ measurements of water levels (USA and India) and storage (South Africa and Spain) as discussed in the **Validation** section of the paper, resulting in a good match between EO and in-situ dataset (r2 values area higher than 0.7 for 68% of reservoirs). However, these types of in-situ measurements are not available globally as open dataset. Global datasets of a high spatio-temporal resolution for small/medium sized reservoirs are scarce. One of the datasets that is has global coverage is GRACE, providing monthly surface mass changes globally. After a detailed analysis of this dataset and comparison with our time series, despite the fact that GRACE is not equivalent to our dataset due to too large spatio-temporal gap between these datasets, we did compare GRACE with our data. Our surface water area time series dataset is derived from satellite imagery of 10-30m resolution while GRACE dataset native resolution is 111319m, resulting in that the latter includes many more water (mass) change effects, including groundwater, rivers, and other mass changes, as well as a mismatch in the phase of the signal (probably due to groundwater flow effects). Fig 2. shows one of the examples where we could find a correlation between our time series and GRACE water thickness estimates while in-situ data results in a perfect match. However, it would still be interesting to explore the GRACE dataset in the context of basin-wide water balance analysis, aggregating multiple waterbodies. This kind of research goes beyond the scope of our paper.

| **Figure 2. Validation of surface water area time series with GRACE dataset for Vaal Dam, South Africa**  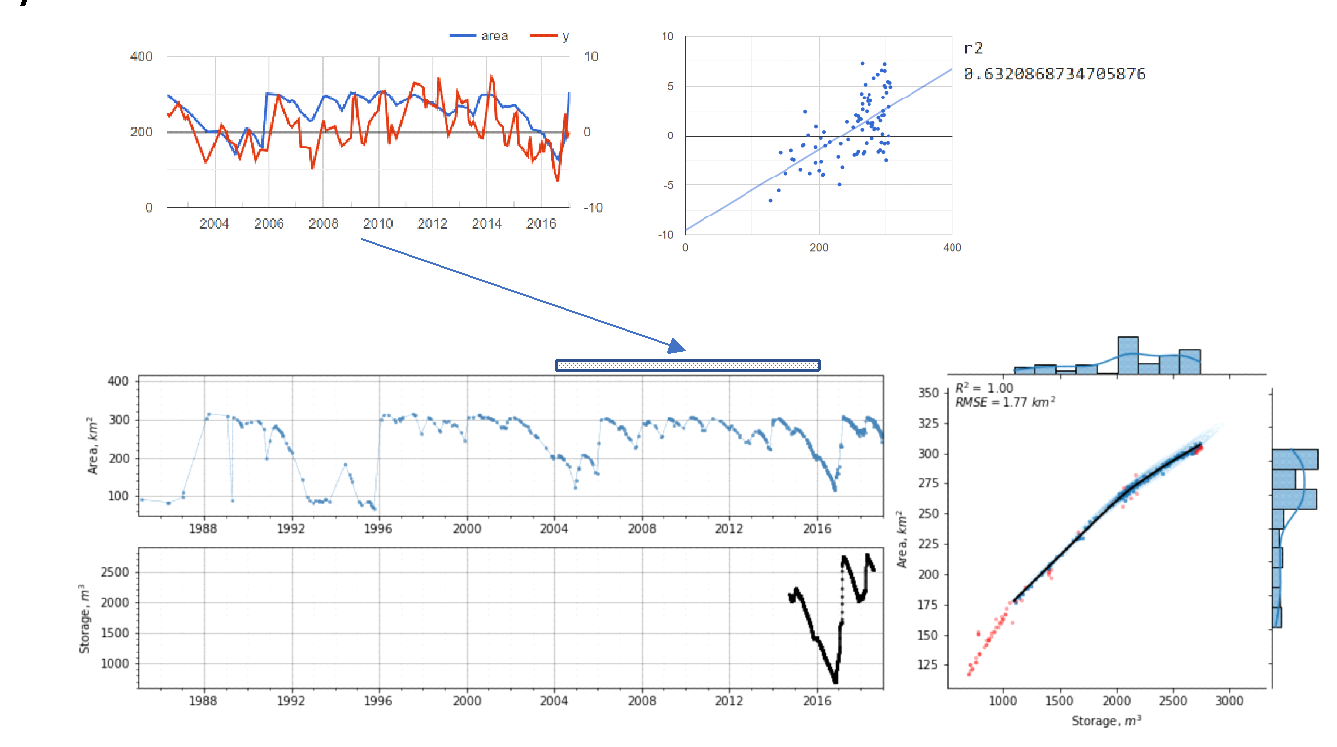  a) comparison of our surface water area time series to GRACE monthly time series of water thickness (averaged to monthly) and b) comparison with in-situ storage estimates    Code: <https://code.earthengine.google.com/81b8d3ce986635a4c1cb4dc73ae239e7> |
| --- |
